# Assessment of the risk factors of duodenogastric reflux in relation to different dietary habits in a Chinese population of the Zhangjiakou area

**DOI:** 10.29219/fnr.v67.9385

**Published:** 2023-10-24

**Authors:** Zhao Peng, Rui Wang, Na Wu, Huiru Gao, Huibin Gao, Duo Li

**Affiliations:** Department of Gastroenterology, The First Affiliated Hospital of Hebei North University, Zhangjiakou, Hebei, China

**Keywords:** duodenogastric reflux, dietary composition, diet, gastrointestinal hormones, risk factors

## Abstract

**Objective:**

To explore the risk factors of duodenogastric reflux (DGR) in relation to different dietary habits.

**Methods:**

A total of 106 patients with symptoms of DGR who underwent electronic gastroscopy from June 2019 to June 2020 were selected and divided into the DGR group (*n* = 33) and the non-DGR group (*n* = 73) according to the diagnosis of bile reflux. Questionnaires were used to collect the basic information and dietary habits of the patients, including age, gender, body mass index, place of residence, comorbidities, dietary composition, salt intake, smoking and drinking consumption. The total bile acid (TBA) and cholesterol (CHO) of the gastric juice were measured using a fully automated biochemical analyser, with an enzyme-linked immunosorbent assay used for the serum cholecystokinin, gastrin and gastrin levels. Univariate analysis and multivariate logistic regression analysis were used to predict the attendant DGR risk factors.

**Results:**

There was no significant difference in age or gender between the DGR and the non-DGR groups (*P* > 0.05). The proportion of patients living in the Bashang region was significantly higher in the DGR group (78.79%) than in the non-DGR group (38.36%) (*P* < 0.05). The levels of TBA and CHO in the gastric juice and the cholecystokinin and gastrin levels in the serum of the DGR group were higher than those in the non-DGR group, while the serum motilin levels were significantly lower in the DGR group than in the non-DGR group (*P* < 0.05). The univariate analysis indicated that the proportion of patients with daily consumption of dairy products and fried foods, a high salt intake and smoking and drinking consumption were significantly higher in the DGR than in the non-DGR group (*P* < 0.05).

**Conclusion:**

The daily consumption of dairy products and a preference for fried food are independent risk factors for the occurrence of DGR (odds ratio ≥ 1, *P* < 0.05).

## Popular scientific summary

- Duodenogastric reflux (DGR) is prone to occur after gastrointestinal and gallbladder surgery, and is associated with the pathogenesis of a variety of upper gastrointestinal diseases, including esophagitis, gastritis, duodenal ulcer and gastric ulcer. It is important to identify the risk factors of DGR for effective dietary prevention and treatment, as well as for digestive function and physical health. By analyzing the dietary habits of patients with DGR, this study found that smoking, drinking, low vegetable intake and preference for high salt food were the influencing factors of DRG, and more dairy products and preference for fried food were the independent risk factors of DGR. BMI and residence were also found to be the risk factors of bile reflux. This study provides the basis and data support for the clinical diagnosis of DRG and bile reflux, and provides an effective scheme for the treatment of DRG through improving personal lifestyle and diet intervention.

Duodenogastric reflux (DGR), also known as bile reflux, is a natural physiological phenomenon that is generally defined as the transport of duodenal content (e.g. bile acid, bile salt, haemolytic lecithin and trypsin) from the duodenum to the stomach (1). Under pathological conditions, duodenal fluid can cause an increase of inflammatory cells in the gastric mucosa, a reduction in parietal cells, the proliferation of mucous cells and morphological changes of the glands, resulting in the destruction of the mucus barrier and direct chemical damage to the gastric surface epithelium (2). DGR can easily occur following gastric surgery, cholecystectomy and pyloroplasty and can also result from gastroduodenal dysmotility (3). Pathological DGR is more harmful to the body. While the clinical prevalence of DGR is not fully understood, there is growing evidence that for approximately 50–86% of patients with gastroesophageal reflux disease (GERD), gastric and duodenal fluids mix during reflux (4). In addition, DGR is associated with the pathogenesis of a variety of upper gastrointestinal diseases, including esophagitis, gastritis, duodenal ulcers and gastric ulcers (5), while studies have demonstrated bile reflux as an independent risk factor for gastric carcinogenesis (6) and hypopharyngeal carcinogenesis (7, 8). Therefore, controlling the occurrence of DGR is of great importance for the digestive function and the health of the body.

To effectively control the occurrence of DGR, an in-depth analysis of the attendant risk factors is required. While DGR is a chronic recurrent reaction involving multiple factors, there exist few studies on the risk factors affecting the development of primary DGR. In China, due to improvements in quality of life and the faster pace of society, people’s dietary structures and habits have undergone major changes (9). Furthermore, geographical vastness and civilisational factors have led to significant differences in dietary structures (10). It is well known that personal habits and dietary structure have a significant impact on digestive health. Meanwhile, GERD involves severe reflux of duodenal content, and a high intake of dairy products and lipids is associated with GERD symptoms (11); however, there is a lack of evidence as to whether dairy and lipid intake directly affects DGR. At present, there are few reports on the relationship between dietary habits and DGR.

Identifying the risk factors for the development of DGR is essential for effective dietary prevention and dietary treatment. This paper presents a cross-sectional study aimed at investigating the association between dietary habits and the risk of DGR by analysing the dietary habits of patients with the condition. The study provides a basis and data support for the clinical diagnosis and dietary treatment of bile reflux.

## Materials and methods

### Study design

A nutritional dietary questionnaire was administered to the study participants who were divided into the DGR and non-DGR groups according to the presence or absence of bile reflux. The diagnosis of bile reflux was made via endoscopy, where yellow-stained gastric mucosa and granular changes in blood vessels, congestion, oedema, tissue fragility, erosion, necrosis and bleeding could be observed (12). The demographic characteristics and clinical information, including gender, age, body mass index (BMI), place of residence, comorbidities, lifestyle habits, dietary structure, gastric fluid composition and gastrointestinal hormones, were analysed and compared between the two patient groups.

### Population

A total of 106 patients with DGR symptoms who underwent electronic gastroscopy from June 2019 to June 2020 (fasting and water prohibition at least 8 h prior to gastroscopy) were selected and divided into the DGR group (*n* = 33) and the non-DGR group (*n* = 73) according to the diagnosis of bile reflux. The inclusion criteria were as follows: (1) persistent abdominal pain, abdominal distension, an obvious burning sensation in the stomach and behind the sternum, acid reflux, bitter taste, nausea and vomiting, loss of appetite and other symptoms in the upper abdomen; (2) patients with the ability to independently comprehend and provide the questionnaire information; (3) patients who have lived in the city for a long time. The exclusion criteria were as follows: (1) patients with partial or total gastric resection; (2) patients with cholecystitis or biliary tract disease; (3) patients with cachexia, ascites, peptic ulcer or malignancy; (4) patients with a history of psychiatric disease; (5) patients with severe heart, brain, kidney, lung or liver diseases; (6) patients with other serious medical diseases resulting in unconsciousness and (7) patients with a *Helicobacter pylori* infection. The study was approved by the ethical review committee of our institution and all the patients included in the study signed the informed consent form for participation.

### Research methods

The questionnaire was used to collect the basic information and the details of the nutritional diet of the patients, including age, gender, BMI (formula: BMI = weight [kg]/height [m^2^]), place of residence, comorbidities (including hypertension, diabetes, hyperlipidaemia, fatty liver), dietary structure (including staple food, vegetables, livestock products, poultry products, soy products, dairy products, fried foods) and salt intake (in this study, we used the dietary survey method to assess salt intake, which involved evaluating the amount of salt consumed by investigating the daily eating habits of individuals). The standard for assessing salt intake was based on one beer bottle cap of salt, which is approximately equivalent to 6 g, smoking and drinking consumption. The contents of total bile acid (TBA) and cholesterol (CHO) in the gastric juice were detected using an automatic biochemical analyser, while the levels of cholecystokinin, motilin and gastrin in the serum of all the participants were detected via a double-antibody sandwich enzyme-linked immunosorbent assay (see Attachment file 1).

To ensure the accuracy of the survey in this study, the questionnaire used in this study was designed by the research team, based on the study aims.

After the first version of the questionnaire was formulated, we invited doctors and hospital administrators to repeatedly discuss and modify the questionnaire. The experts agreed that the final version of the questionnaire was effective, reliable and scientific and could be used for its intended purpose.

In addition, the SPSS Statistics (v.21.0) software was used to test the reliability and validity of the questionnaire. The Cronbach’s coefficient was 0.81. The Kaiser-Meyer-Olkin (KMO) value was 0.60; *P* < 0.001 passed the Bartlett’s Test of Sphericity and the cumulative variance interpretation rate value was 64.04%.

### Statistical analysis

The sample size was calculated via PASS 15.0 to obtain *n* = 106, while all the data were statistically analysed using the SPSS Statistics software. The measurement data that followed a normal distribution were expressed as mean ± standard deviation, with a one-way analysis of variance test used for intergroup comparisons. The variables that followed a skewed distribution were expressed as the median (Q1, Q3), and the differences between the groups were compared using the Kruskal-Wallis test or the Mann-Whitney U test. The count data were expressed as the number of cases/percentage (*n*/%) and were tested using a chi-square test or Fisher’s exact test. Logistic regression analysis was adopted to analyse the risk factors of DGR in relation to different eating habits. A *P-*value of <0.05 indicated that the differences were statistically significant.

## Results

### General information of participants

The demographic characteristics of the participants in both groups are shown in Table 1. There were 33 patients in the DGR group, with an average age of 53.97 ± 14.98 years, including 14 males and 19 females. There were 73 patients in the non-DGR group, with an average age of 49.84 ± 17.76 years, including 41 males and 32 females. There were no significant differences in sex ratio or age between the two groups (*P* > 0.05). Compared to the non-DGR group, the BMI of the DGR group was larger, and the difference was statistically significant (*P* < 0.05). In addition, the proportion of patients living in the Bashang region was higher in the DGR (78.79%) than in the non-DGR group (38.36%), and the difference was statistically significant (*P* < 0.05). These results suggest that BMI and place of residence could be risk factors for the occurrence of bile reflux.

**Table 1:** Demographic characteristics of patients

Item	DGR group (*n* = 33)	Non-DGR group (*n* = 73)	*t*/X^2^	*P*
Age (years)	53.97 ± 14.98	49.84 ± 17.76	1.162	0.248
Male	14 (42.42%)	41 (56.16%)		
Female	19 (57.58%)	32 (43.84%)		
BMI	27.57 ± 4.46	23.57 ± 1.69	6.713	<0.001
Place of residence [*n* (%)]			14.866	<0.001
Bashang region	26 (78.79%)	28 (38.36%)		
Baxia region	7 (21.21%)	45 (61.64%)		

### Comparison of gastric juice components and gastrointestinal hormones between the two patient groups

Disturbances in gastrointestinal hormone levels may contribute to the development of bile reflux. The results for the TBA and CHO content in the gastric juice indicated that both levels were considerably higher in the DGR than in the non-DGR group, and the difference was statistically significant (*P* < 0.05). The results for the serum gastrointestinal hormone levels indicated that the content of cholecystokinin and gastrin were considerably higher in the DGR than in the non-DGR group (cholecystokinin: 50.32 ± 21.55 vs. 38.33 ± 18.71; gastrin: 98.60 ± 34.06 vs. 83.63 ± 34.37; *P* < 0.05). The level of motilin was significantly lower in the DGR than in the non-DGR group (240.38 ± 94.16 vs. 303.74 ± 95.94; *P* < 0.05). The results are shown in Table 2.

**Table 2:** Comparison of gastric juice components and gastrointestinal hormones between two groups of patients

Item	DGR group (*n* = 33)	Non-DGR group (*n* = 73)	*t*	*P*
Total bile acids (μmol/L)	11.24 ± 2.17	8.18 ± 0.77	10.676	<0.001
Cholesterol (mmol/L)	5.39 ± 0.97	4.39 ± 0.95	4.956	<0.001
Cholecystokinin (pg/ml)	50.32 ± 21.55	38.33 ± 18.71	2.911	0.040
Gastrin (pg/ml)	98.60 ± 34.06	83.63 ± 34.37	2.082	0.040
Motilin (pg/ml)	240.38 ± 94.16	303.74 ± 95.94	−3.167	0.002

### Comparison of dietary composition between the two groups

The dietary composition of the two groups of patients was further analysed, with the results presented in Table 3. Here, it was found that there was a significant difference in staple food composition and vegetable intake in the DGR compared to the non-DGR group (*P* < 0.05), while the vegetable, livestock and poultry product types were not significantly different between the two groups (*P* < 0.05). As Table 3 shows, the staple food of the DGR group was dominated by pasta (63.64%) and the intake of vegetables was lower compared to the non-DGR group.

**Table 3:** Comparison of dietary composition of two groups of patients

Item	DGR group (*n* = 33)	Non-DGR group (*n* = 73)	t/X^2^	*P*
Staple food composition [*n* (%)]			8.041	0.018
Rice	11 (33.33%)	45 (61.64%)		
Pasta	21 (63.64%)	25 (34.25%)		
Coarse food grain	1 (3.03%)	3 (4.11%)		
Vegetable type [*n* (%)]			0.039	0.998
Leafy vegetables	23 (69.70%)	51 (69.86%)		
Rhizome	6 (18.18%)	14 (19.18%)		
Melon and eggplant	2 (6.06%)	4 (5.48%)		
Beans	2 (6.06%)	4 (5.48%)		
Vegetable intake [*n* (%)]			7.659	0.022
≤200 g	14 (42.42%)	15 (20.55%)		
200–500 g	19 (57.58%)	51 (69.86%)		
≥500 g	0 (0%)	7 (9.59%)		
Animal product type [*n* (%)]			2.321	0.509
Pork	26 (78.79%)	59 (80.82%)		
Beef	3 (9.09%)	6 (8.22%)		
Lamb	3 (9.09%)	8 (10.96%)		
Internal organs	1 (3.03%)	0 (0%)		
Poultry products [*n* (%)]			0.339	0.561
Poultry meat	32 (96.97%)	72 (98.63%)		
Internal organs	1 (3.03%)	1 (1.37%)		

### Comparison of living habits of the two groups of patients

The statistical analysis of the living habits of the two groups of patients indicated that the proportion of patients with daily consumption of dairy products (87.88%) was significantly higher in the DGR than in the non-DGR group (24.66%) (*P* < 0.001). The daily salt intake in the DGR group was also significantly higher than in the non-DGR group (*P* < 0.05). In addition, the proportion of patients in the DGR group who had a preference for fried foods was 72.73%, which was higher than in the non-DGR group (20.55%), and the difference was statistically significant (*P* < 0.001). There was no significant difference in the daily consumption of soy products between the two groups (*P* > 0.05). Compared with the non-DGR group, patients in the DGR group smoked more (7.41 ± 1.28 vs. 3.21 ± 1.36) and had a higher level of alcohol consumption (69.45 ± 3.77 vs. 31.27 ± 4.11), and the difference was statistically significant (*P* < 0.05) (see Table 4).

**Table 4:** Comparison of living habits between the two groups

Item	DGR group (*n* = 33)	Non-DGR group (*n* = 73)	*t*/X^2^	*P*
Daily consumption of soy products [*n* (%)]			0.44	0.834
Yes	16 (48.48%)	37 (50.68%)		
No	17 (51.52%)	36 (49.32%)		
Daily consumption of dairy products [*n* (%)]			36.81	<0.001
Yes	29 (87.88%)	18 (24.66%)		
No	4 (12.12%)	55 (75.34%)		
Salt intake [*n* (%)]			6.194	0.045
≤6 g	7 (21.21%)	34 (46.58%)		
6–20 g	23 (69.70%)	35 (47.95%)		
≥20 g	3 (9.09%)	4 (5.48%)		
Smoking consumption (cigarettes/day)	7.41 ± 1.28	3.21 ± 1.36	4.710	0.030
Drinking consumption (g/day)	69.45 ± 3.77	31.27 ± 4.11	5.763	0.016
Yes	18 (54.55%)	22 (30.14%)		
No	15 (45.45%)	51 (69.86%)		
Frying [*n* (%)]			26.61	<0.001
Yes	24 (72.73%)	15 (20.55%)		
No	9 (27.27%)	58 (79.45%)		

### Comparison of comorbidities between the two groups

The *P*-values in the comparison between the two groups were all greater than 0.05 in terms of the following: hypertension (DGR group = 21.21%, non-DGR group = 10.96%; *P* = 0.161); hyperlipidaemia (DGR group = 24.24%, non-DGR group = 13.70%; *P* = 0.181); diabetes (DGR group = 3.03%, non-DGR group = 2.74%; *P* = 0.933) and fatty liver (DGR group = 24.24%, non-DGR group = 26.03%; *P* = 0.845) (see Table 5). It can thus be concluded that the above factors are not risk factors for the occurrence of DGR.

**Table 5:** Comparison of comorbidities between the two groups of patients

Item	DGR group (*n* = 33)	Non-DGR group (*n* = 73)	t/X^2^	*P*
Hypertension [*n* (%)]			1.967	0.161
Yes	7 (21.21%)	8 (10.96%)		
No	26 (78.79%)	65 (89.04%)		
Hyperlipidemia [*n* (%)]			1.792	0.181
Yes	8 (24.24%)	10 (13.70%)		
No	25 (75.76%)	63 (86.30%)		
Diabetes [*n* (%)]			0.007	0.933
Yes	1 (3.03%)	2 (2.74%)		
No	32 (96.97%)	71 (97.26%)		
Fatty liver [*n* (%)]			0.038	0.845
Yes	8 (24.24%)	19 (26.03%)		
No	25 (75.76%)	54 (73.97%)		

### Logistic regression analysis of risk factors for bile reflux

Factors with a *P*-value < 0.05 in the univariate analysis were subjected to logistic regression analysis. This included age, residence, TBA, CHO, cholecystokinin, gastrin, motilin, staple food composition, vegetable intake, daily consumption of dairy products, salt intake, fried food consumption, smoking and alcohol consumption. The results are shown in Table 6. Here, TBA level [odds ratio (OR) = 1.751, 95% confidence interval (CI): (1.185, 2.803), *P* = 0.010], motilin [OR = 0.985, 95% CI: (0.975, 0.992), *P* = 0.001], the daily consumption of dairy products [OR = 15.680, 95% CI: (3.173, 102.8), *P* = 0.002] and fried foods [OR = 10.150, 95% CI: (2.181, 60.73), *P* = 0.005] were the independent factors related to DGR.

**Table 6:** Logistics regression analysis of risk factors for bile reflux

Item	B Wald	Standard error	*P*	OR	95% CI
BMI	−0.079	0.098	0.421	0.924	0.754 ~ 1.112
Place of residence	−1.544	1.032	0.135	0.214	0.024 ~ 1.448
Total bile acids	0.560	0.217	0.010	1.751	1.185 ~ 2.803
Cholesterol	−0.001	0.346	0.998	0.999	0.502 ~ 2.007
Cholecystokinin	0.009	0.018	0.619	1.009	0.974 ~ 1.047
Gastrin	−0.013	0.011	0.231	0.987	0.965 ~ 1.007
Motilin	−0.015	0.005	0.001	0.985	0.975 ~ 0.992
Staple food composition	−0.446	0.419	0.286	0.640	0.260 ~ 1.387
Vegetable intake	0.121	0.657	0.854	1.129	0.306 ~ 4.192
Daily consumption of dairy products	2.752	0.871	0.002	15.680	3.173 ~ 102.8
Salt intake	−0.319	0.588	0.588	0.727	0.215 ~ 2.243
Smoking consumption	0.325	0.664	0.679	1.340	0.461 ~ 5.396
Drinking consumption	1.011	0.749	0.203	2.741	0.633 ~ 13.231
Frying	2.317	0.832	0.005	10.150	2.181 ~ 60.73

## Discussion

The results of this study demonstrate that the daily consumption of dairy products and a preference for fried food are independent risk factors for DGR. Previous studies have confirmed that slight levels of DGR may be physiologically present in the stomach after feeding and fasting (13). Bile is considered a major duodenal component. It is produced in the liver and stored in the gallbladder, with its main role being to assist in the digestion and absorption of lipids (14). When bile, pancreatic juice and duodenal juice flow back into the gastroesophageal junction, the mucus barrier is often destroyed, resulting in damage and inflammation of the normal mucosa of the gastroesophagus (15). Existing studies reported bile reflux as being closely related to the development of upper gastrointestinal inflammation and ulcers (5, 8, 16, 17). In addition, DGR contributes to intestinal metaplasia and the subsequent development of gastric cancer (18). Elsewhere, an observational cross-sectional study demonstrated that bile reflux is an independent risk factor for gastric precancerous lesions and gastric cancer (6). Recent research evidence suggests that DGR is also an independent risk factor for NF-κB-mediated hypopharyngeal cancer (7, 8). In addition, when combined with gastric acid, the bile and pancreatic enzymes in DGR can cause damage to tooth enamel (19). All of the above findings indicate that both physiological and pathological bile reflux can be harmful to the organism; accordingly, it is important to investigate the attendant influencing factors and control the occurrence of bile reflux for improved human health.

Studies have revealed that DGR commonly occurs following cholecystectomy (20), pyloroplasty (21) and gastric surgery (22); however, the present study focused on the risk of primary bile reflux occurrence. In fact, DGR is a chronic recurrent physiological and pathological response with multifactorial involvement, and the exact pathogenesis of primary DGR is not fully understood. Existing studies have speculated that age may be an influential factor in the occurrence of DGR, with the results indicating that primary DGR is more common in the 21–30 and 50–80-year age groups (23, 24). A study involving children with primary DGR found that these children were significantly older than the healthy controls (2). In the present study, there was no significant difference in the average age between the DGR and the non-DGR group, suggesting that there may be no significant difference in the incidence of bile reflux among adults aged 21–87 years.

The effect of gender on the occurrence of DGR remains a controversial topic. Barakat et al. (23) reported that DGR may be more common in women, while Mercan et al. (20) reported no significant differences in gender between their DGR and non-DGR groups. In the present study, there was no statistically significant difference in the ratio of gender between the DGR and the non-DGR groups, supporting the findings obtained by Mercan et al.

Interestingly, the present study found that the proportion of patients living in Bashang was higher in the DGR group than in the non-DGR group (*P* < 0.05), suggesting that place of residence could be one of the risk factors for bile reflux. The patients in this study live in an area characterised by the continental monsoon climate of East Asia, with the topography high in the northwest and low in the southeast. The Yinshan Mountains divide the city into two natural areas, namely, the Bashang and Baxia regions. Here, different eating habits have been adopted due to the different geographical locations. For example, people living in the Bashang area tend to eat twice a day with long intervals, have a preference for pasta and fatty meat, often eat pickled and fried foods, tend to eat fewer vegetables and fruits and have a habit of drinking alcohol for breakfast. Meanwhile, with population migration and the popularisation of dietary health knowledge, the dietary structure of the people in the Baxia area has changed significantly. Here, the people tend to eat three meals a day, choose rice as their main staple food, consume less meat and salt, eat more vegetables and consume less alcohol than those in the Bashang region. The difference in the incidence of DGR between the patients living in the Bashang region and those in the Baxia region implies the potential influence of lifestyle habits and dietary structure on the occurrence of DGR.

Smoking, drinking and the intake of dairy products, lipids and salt in the DGR group were higher than in the non-DRG group (*P* < 0.05). Existing studies have demonstrated that smoking and alcohol abuse can induce DGR (25). Meanwhile, studies on GERD have reported that fried, acidic and spicy foods are triggers of GERD, with high-fat, high-sugar diets among the attendant risk factors (26), and a high-fat intake also increasing the observation of reflux symptoms (27). Furthermore, DGR is an important factor affecting the occurrence of GERD (28); this suggests that dietary structure may affect GERD by affecting the occurrence of DGR.

The present study also found significant differences in staple food composition and vegetable intake between the two groups (*P* < 0.05). Scholars have found rice-based food to have good absorption and capable of improving the symptoms of functional gastrointestinal diseases (29), while the dietary fibre in vegetables can also improve gastrointestinal diseases (30). The results of the logistic regression analysis conducted for the present study indicated that the daily consumption of dairy products and a preference for fried foods were independent risk factors for DGR. These results suggest that the differences in dietary habits due to different geographical locations are relevant factors affecting the occurrence of DGR, and also highlight the importance of smoking and drinking cessation and adopting a balanced diet.

Mechanistically, DGR can be caused by any factor that leads to gastrointestinal motility disorders and anatomical abnormalities, such as major gastrectomy (31), disruption of gastrointestinal hormone secretion (32) and autonomic dysfunction (33). Among them, disorders of gastrointestinal hormone secretion have a greater impact on primary DGR. The primary bile acids, cholic acid and chenodeoxycholic acid, are synthesised in the hepatocytes, conjugated with either glycine or taurine and then transported from the liver to the gall bladder for storage (34). During the consumption of a meal, bile fluid flows into the intestinal duct, where the bile acids emulsify and solubilise lipid-soluble nutrients to facilitate dietary digestion and absorption. The gut hormone, cholecystokinin, is synthesised by the small intestinal enteroendocrine cells, and its major effect in the gastrointestinal tract is to induce gall bladder contraction and the delivery of bile in the small intestine (35, 36). Meanwhile, motilin is released from the duodenum to increase gastric emptying and gastric motor activity in denervated gastric pouches (37). Gastrin is a gastrointestinal motility hormone secreted by the gastric antrum and duodenum. It has specific functions, such as regulating the tension of the lower oesophageal sphincter, and can also promote intestinal motility, mainly by enhancing intestinal smooth muscle contraction (38). Zhao et al. (39) reported that elevated levels of bile acids in gastric juice are associated with bile reflux gastritis in humans. Sirchak et al. (40) reported that cholecystokinin levels are significantly higher in patients with GERD than in healthy individuals. Motilin has been reported to be low in the serum of patients with GERD (41, 42). In a recent report, lower levels of gastrin-17 were detected in patients with typical GERD symptoms (43). However, the direct relationship between gastrointestinal hormone balance and primary DGR has not yet been reported.

Given that GERD is often accompanied by the development of bile reflux, the characterisation of various gastrointestinal hormones in GERD implies a potential correlation between gastrointestinal hormones and DGR. In the present study, the gastric TBA, serum cholecystokinin and gastrin levels were found to be significantly higher in the DGR than in the non-DGR group, while the serum motilin in the DGR group was significantly lower. The logistic regression analysis suggested that TBA and motilin could be used as predictors of DGR. An increase in cholecystokinin may facilitate sphincter relaxation, bile excretion and common bile duct pressure regulation, while an increase in gastrin may relax the pyloric sphincter, exacerbate the bile reflux and cause elevated bile acid levels in the gastric juice. Low levels of motilin tend to result in relaxation of the gastrointestinal smooth muscle, decreased gastric motility and a prolonged gastric emptying time, leading to increased retention of refluxed duodenal fluid in the gastric lumen and providing favourable conditions for bile damage to the gastric mucosa.

This study also found differences in the levels of gastrointestinal hormones between the patients living in the Bashang region and those in the Baxia region, who have different dietary habits, suggesting that the differences in lifestyle habits and dietary structure may exert an influence on the occurrence of DGR by regulating the balance of various gastrointestinal hormones. However, the specific underlying mechanism of this requires further exploration.

This study has some limitations. Firstly, the research represents a case-control study, the sample size was small and the geographical scope of the population selection was limited to a local city; as such, it is necessary to expand the geographical scope of the topic or conduct a multicentre study. Secondly, the influencing factors included in the analysis were not fully comprehensive. For example, night-time eating, particularly before bed, and sweet food intake should also be considered. Finally, intervention trials are needed to further verify the conclusions, as it concerns exploring the effect of dietary therapy on bile reflux.

## Conclusion

Differences in lifestyle habits and dietary structure, based on geographic location, can affect the occurrence of DGR. The daily consumption of dairy products and a preference for fried food are independent risk factors for the development of the disease. Dietary differences may affect the gastrointestinal hormone balance to influence the occurrence of DGR. In daily life, DGR can be treated by improving the individual’s lifestyle and through dietary intervention.

## Declarations

### Availability of data and materials

All data generated or analysed during this study are included in this article.
